# Manufacturing of primary CAR-NK cells in an automated system for the treatment of acute myeloid leukemia

**DOI:** 10.1038/s41409-023-02180-4

**Published:** 2024-01-22

**Authors:** Nawid Albinger, Sabine Müller, Julia Kostyra, Jan Kuska, Sarah Mertlitz, Olaf Penack, Congcong Zhang, Nina Möker, Evelyn Ullrich

**Affiliations:** 1 Goethe University, Department of Pediatrics, Experimental Immunology and Cell Therapy, Frankfurt am Main, Germany; 2 Frankfurt Cancer Institute, Goethe University, Frankfurt am Main, Germany; 3 Miltenyi Biotec B.V. & Co. KG, Bergisch Gladbach, Germany; 4 Charité, Universitätsmedizin Berlin, corporate member of Freie Universität Berlin and Humboldt-Universität zu Berlin, Department of Hematology, Oncology and Tumorimmunology, Berlin, Germany; 5 German Cancer Consortium (DKTK), partner site Berlin, Berlin, Germany; 6 German Cancer Consortium (DKTK) partner site Frankfurt/Mainz, Frankfurt am Main, Germany

**Keywords:** Immunotherapy, Acute myeloid leukaemia

## Abstract

Acute myeloid leukemia (AML) still constitutes a dreadful disease with limited therapeutic options. Chimeric antigen receptor (CAR)-modified T cells struggle to target AML partly due to a lack of true AML-exclusive antigens and heterogeneity of the disease. Natural killer (NK) cells possess a high intrinsic killing capacity against AML and might be well suited for the treatment of this disease. However, the generation of primary CAR-NK cells can be difficult and time consuming. Therefore, robust systems for the generation of high numbers of CAR-NK cells under GMP conditions are required. Here we report on the automated generation of high numbers of primary CD33-targeting CAR-NK cells using the CliniMACS Prodigy^®^ platform. Automated-produced CD33-CAR-NK cells showed similar phenotype and cytotoxicity compared to small-scale-produced CD33-CAR-NK cells in vitro and were able to strongly reduce leukemic burden in an OCI-AML2 NSG-SGM3 xenograft mouse model in vivo following a cross-site shipment of the cell product. This technology might be well suited for the generation of primary CAR-modified NK cells for a broad range of targets and could facilitate clinical transition.

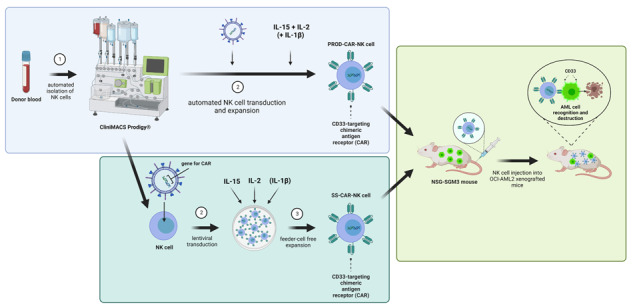

## Introduction

Chimeric antigen receptor (CAR)-modified T cells already demonstrated outstanding results for the treatment of B cell neoplasms such as CD19^+^ B cell acute lymphoblastic leukemia as well as B cell maturation antigen (BCMA)^+^ multiple myeloma which led to the approval of multiple drugs by the U.S. Food & Drug Administration (FDA) as well as the European Medicines Agency (EMA) [1–4]. However, CAR-T cells still face major limitations such as the necessity to be applied in an autologous manner, since allogeneic T cells might carry the risk for major side effects such as graft-versus-host-disease [5, 6]. Furthermore, CAR-T cells might possess limited efficacy against T cell resistant malignancies, which lack cancer-exclusive antigens. Natural killer (NK) cells on the other hand do not cause graft-versus-host-disease and recognize malignant cells in an antigen-independent manner through an intrinsic killing capacity which is orchestrated by a broad range of activating and inhibitory cell-surface receptors [7]. Therefore, CAR-NK cells might be suited to treat CAR-T cell resistant malignancies and offer the potential to be applied as a so-called off-the-shelf product, which could drastically reduce costs and enable fast availability of a CAR-immune cell therapy. The generation of CAR-modified NK cell lines such as CAR-NK-92 cells appears feasible but possesses problems such as the necessity to irradiate CAR-NK-92 cells prior to infusion which can limit proliferation and functionality of these cells in vivo [8–12]. Therefore, the usage of primary peripheral blood-derived NK cells in patients is highly desirable. Yet, their generation still remains challenging as primary NK cells proliferate slower and possess a shorter lifetime compared to primary T cells ex vivo [13, 14]. Hence, one crucial step to enable the generation of clinical CAR-NK cell products is to establish robust methods for the generation of high numbers of primary CAR-NK cells under *Good Manufacturing Practice* (GMP)-conditions [15–17].

In previous work, we were able to show the generation of effective CD33-targeting CAR-NK cells for the treatment of acute myeloid leukemia (AML) [18], a disease for which no CAR-T cell therapy is available yet, and which demonstrates dismal overall survival rates especially for elderly patients [19, 20]. While use of a CliniMACS Prodigy^®^ device was already shown for the generation of CAR-T [21–24] and for the expansion of untransduced NK cells [25], this is the first report on the automated generation of CD33-CAR-NK cells using the CliniMACS Prodigy^®^ platform resulting in a proof-of-concept evaluation in a xenograft AML model in vivo. In this preclinical study, Prodigy-produced (PROD)-CAR-NK cells showed a similar functionality compared to small-scale-produced (SS)-CAR-NK cells from the same donor in vitro and in an OCI-AML2 NSG-SGM3-xenograft mouse model after a multiple-hour transportation in cooled formulation solution.

## Methods

### Isolation of NK cells

NK cells were isolated from leukapheresis, acquired from healthy volunteer donors. Subsequently, automated NK cell separation was performed by immunomagnetic selection via CD3 depletion using CliniMACS Prodigy^®^ LP-3-56 followed by an CD56 enrichment using CliniMACS Prodigy^®^ PD-56 Engineering. NK cell purity was determined as described earlier [18]. This project was approved by the Ethics Committee of the Goethe University Frankfurt, Germany (approval no. 274/18 and 329/10). Peripheral blood apheresis products from healthy donors were obtained from the Institute for Transfusion Medicine of Hannover Medical School (MHH) after donors’ written informed consent. The protocol was approved by the ethics committee of Hannover Medical School.

### CAR construction and lentiviral vector production

A second-generation CD33-targeting CAR which incorporates the My96 scFv sequence, which has been used in the immunoconjugate AVE9633, was constructed as described earlier [26]. In short, the My96 scFv was combined in frame with CD8 hinge and transmembrane domain, 4-1BB/CD137 co-stimulatory domain, and CD3ζ activation domain. A leader peptide derived from granulocyte-macrophage colony-stimulating factor receptor unit α (GM-CSFRα) was included to facilitate CAR cell surface expression. Third-generation self-inactivating baboon envelope-pseudotyped lentiviral vectors (BaEV-LVs) were produced by transient transfection into HEK293T cells using MACSfectin™ (Miltenyi Biotec, Bergisch Gladbach, Germany) [18, 27].

### CAR-NK cell manufacturing

Two days after isolation, NK cells were transduced with lentiviral particles either as described earlier for the small-scale production [18] or using the CliniMACS Prodigy^®^ device. Briefly, purified NK cells were cultivated with NK MACS GMP Medium supplemented with 5% human AB serum, MACS GMP recombinant human IL-2, MACS GMP recombinant human IL-15 and additionally IL-1β for activation. On day 2 of culture, transduction was performed by addition of pre-mixed lentiviral vector particles with 1.25 µg/mL Vectofusin-1 to NK cells followed by spinoculation for 2 h at 32 °C and 400 *g*. NK cells were washed at day 3 to remove excess lentiviral vector. Subsequently, NK cells were cultured until day 14 using NK MACS GMP Medium supplemented with 5% human AB serum, as well as MACS GMP recombinant human IL-2 and IL-15. The NK cells in small scale were cultured according to the same scheme. After the first culture week, small-scale cells were split in a 1:2 ratio on day 9, 10 and 13.

### Flow cytometry analysis of transduced NK cells

During culture, the NK cell population was monitored by flow cytometry using a standardized in process control (IPC) and quality control (QC) strategy. The NK cell purity was verified using the NK cell composition panel including the fluorochrome-conjugated antibodies anti-CD45-VioBlue (clone REA747, Miltenyi Biotec), anti-CD14-VioGreen (clone REA599, Miltenyi Biotec), anti-TCRαβ-FITC (clone REA652, Miltenyi Biotec), anti CD3-PE (clone SK7, BioLegend, San Diego, CA, USA), anti-CD56-APC (clone REA196, Miltenyi Biotec) and anti-CD19-APC-Vio770 (clone REA675, Miltenyi Biotec).

CAR expression was analyzed by using a CD33-CAR Detection Reagent which contained a recombinantly expressed fusion protein that consisted of the extracellular domains of human CD33 and a specifically mutated human IgG1-Fc region connected to biotin, and secondary a biotin-targeting antibody, anti-biotin PE (clone REA746) (both Miltenyi Biotec).

NK cell transduction efficacy was determined through the NK cell transduction panel containing anti-CD45-VioBlue (clone REA747, Miltenyi Biotec), anti-CD14-VioGreen (clone REA599, Miltenyi Biotec), anti-CD33-CAR Detection Reagent-Biotin (Miltenyi Biotec), anti-Biotin-FITC (clone Bio3-18E7, Miltenyi Biotec), anti CD3-PE (clone SK7, BioLegend) and anti-CD56-APC (clone REA196, Miltenyi Biotec).

For the phenotypical analysis of untransduced (UTD)- and CAR-NK cells, fluorochrome-conjugated antibodies anti-NKG2A-PE (clone Z199, Beckman Coulter, Brea, CA, USA), anti-NKG2C-AF488 (clone 134591, R&D Systems, Minneapolis, MN, USA), anti-CD3-PerCP (clone UCHT-1, BioLegend), anti-CD57-BV421 (clone NK-1), anti-CD69-BV605 (clone FN50) and anti-CD16-PE-CF594 (clone 3G8) (all BD Biosciences, Franklin Lakes, NJ, USA) were used.

All stainings were performed using FcR Blocking Reagent (Miltenyi Biotec) or unspecific hIgG (Kiovig or Intratect®, University Hospital Frankfurt, Frankfurt am Main, Germany) to increase the specificity of immunofluorescent staining and 7-amino-actinomycin D (7AAD) (Miltenyi Biotec) for the exclusion of dead and apoptotic cells from flow cytometric analysis.

### In vitro cytotoxicity assay

SS- and PROD-produced UTD-NK cells as well as SS-/PROD-produced CAR-NK cells were co-incubated for four hours with OCI-AML2 cells at an effector-to-target (E:T) ratio of 1:5. OCI-AML2 cells were previously labeled using Cell Trace CFSE proliferation kit (Thermo Fisher Scientific, Waltham, MA, USA). Subsequently, cells were stained with 4′,6-diamidino-2-phenylindole (DAPI) (AppliChem, Darmstadt, Germany) and the viability of target cells was analyzed using a BD FACSCelesta^TM^ device (BD Biosciences).

### In vivo analysis of PROD-CAR-NK cells in an OCI-AML2-xenograft NSG-SGM3 mouse model

In vivo functional comparison of SS- and PROD-produced CAR-NK cells was performed in our established OCI-AML2-xenograft NSG-SGM3 mouse model as described earlier [18]. OD.Cg-Prkdcscid Il2rgtm1Wjl Tg(CMV-IL3,CSF2,KITLG)1Eav/MloySzJ (NSG-SGM3) mice were obtained from The Jackson Laboratory, Bar Harbor, ME, USA (JAX stock No.: #013062 (NSG-SGM3)) [28]. Mice were held under standardized pathogen free conditions with adequate access to food and water. Experiments were approved by the Regierungspräsidium Darmstadt, Germany.

### Bone marrow sectioning and analysis

Mouse femurs and tibiae were isolated and incubated in a series of 4% PFA, EDTA- and sucrose-solutions before they were embedded in gelatin. 7 µm-sections of bones were generated on the CryoStar™ NX70 cryostat (Thermo Fisher Scientific). OCI-AML2 cells were detected by their GFP-expression. For nuclear counterstaining DAPI (Sigma-Aldrich, St. Louis, MO, USA) was used.

### In vivo cytokine release

Cytokine levels in sera of mice were determined three days pre OCI-AML2 cell injection and one day post therapy initiation (day 4 post AML cell injection) using a BD™ Cytometric Bead Array (CBA; BD Biosciences) according to manufacturer’s instructions. BD™ CBA Flex Sets were used to measure the cytokine concentrations. Data were obtained using a BD FACSVerse™ Bioanalyzer. For data analysis the FCAP Array software (v3.0.1; BD Biosciences) was used.

### Statistical analysis

For statistical analysis, a normal distribution for NK cell functionality was assumed due to the deployment of healthy donors as NK cell source. For the animal experiments, also a normal distribution could be assumed. Accordingly, data were analyzed by two-tailed, unpaired Student’s *t* test or Mann–Whitney-test and defined as significant when *p* < 0.05. Statistical analysis was performed using GraphPad PRISM version 9 (GraphPad Software, Inc., San Diego, CA, USA).

## Results

**Fig. 1:**
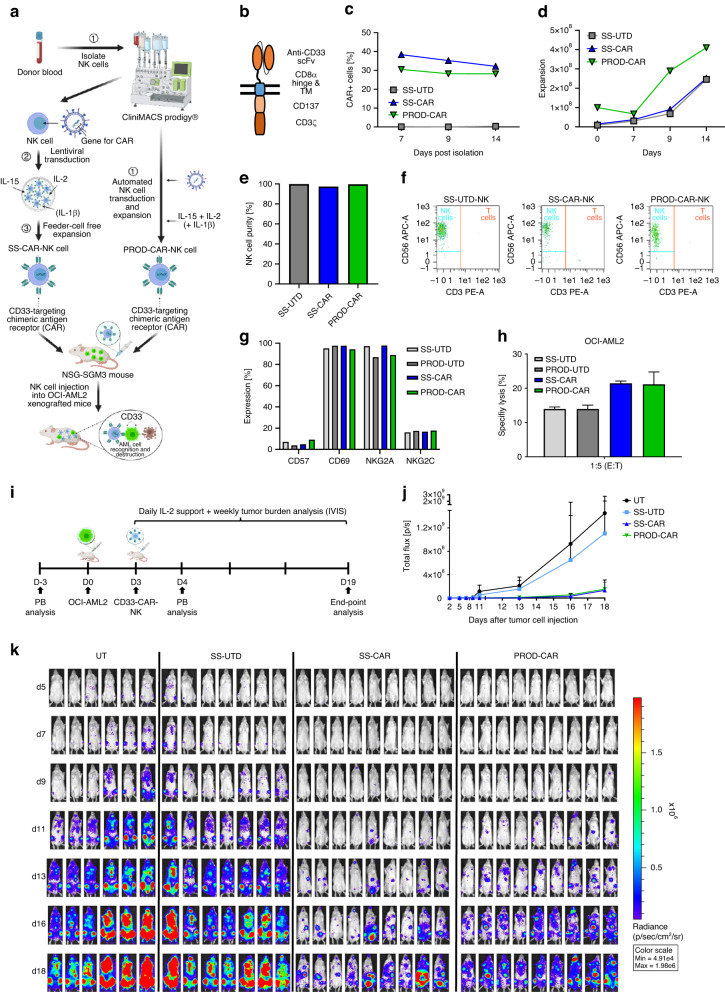
Small-scale (SS-) and CliniMACS Prodigy® (PROD-) produced CD33-CAR-NK cells show similar anti-leukemic effects. **a** Scheme of the experimental setting to compare SS-CAR-NK and PROD-CAR-NK cells in vivo. **b** Scheme of the second-generation CAR-construct, which was incorporated into the NK cells. CAR-expression (**c**), NK cell expansion (**d**) as well as NK cell purity (**e**) and CD3^+^ T cell contamination (**f**) at day 14. **g** Flow-cytometry analysis of cell surface expression of different cell surface markers on SS-CAR-NK and PROD-CAR-NK cells. **h** SS-CAR-NK and PROD-CAR-NK cell in vitro cytotoxicity assay against CD33^+^ OCI-AML2 cells at low effector-to-target-ratio. Cells were co-cultivated for 4 h and dead AML cells quantified as DAPI^+^ by flow cytometry. Shown is one experiment with technical triplicates. Mean ± SD. **i** Scheme of the in vivo comparison of SS-CAR-NK and PROD-CAR-NK cells in an OCI-AML2 NSG-SGM3 xenograft mouse model. Mice received a single dose of 1 ×10^7^ NK cells at day 3 post AML cell injection followed by subcutaneous treatment with IL-2. **j** Total flux analysis (photons/second) as well as bioluminescence images (BLI) (**k**) of differently treated OCI-AML2 (luciferase^+^) NSG-SGM3 xenografted mice over time after subcutaneous injection of luciferin (*n* = 6–9 mice/per group).

With the aim to automatically generate CD33-CAR-NK cells and evaluate their functionality, NK cells were isolated automatically from one leukapheresis product using a CliniMACS Prodigy^®^ device. Subsequent NK cell activation, transduction and expansion was performed either manually in a small-scale setting (SS-UTD/CAR-NK) or using the CliniMACS Prodigy^®^ (PROD-UTD/CAR-NK) with the PD-56 Engineering process (Fig. 1a). The second-generation CAR-construct, which was incorporated into the NK cells, consisted of an extracellular CD33-specific single-chain variable fragment, a CD8α hinge and transmembrane domain, as well as an intracellular CD137 (4-1BB) costimulatory and CD3ζ signaling domain (Fig. 1b). In brief, purified NK cells were activated and expanded in the presence of human IL-2, IL-15 and IL-1β and lentiviral transduction was performed on day 2 followed by an expansion phase as described in the *Methods* section. At the end of the manufacturing process, the quality of the final CAR-NK cell products was assessed. After 14 days of culture, a transduction efficiency of 28.17% was achieved in the Prodigy-process, while the small-scale production resulted in 32.14% CAR-expressing NK cells (Fig. 1c). The NK cell expansion was 4.1-fold in the Prodigy-process, with a starting culture volume of 70 ml and a final culture volume limited to 250 ml and resulted in 1.15 × 10^8^ total CAR-expressing NK cells. In the small-scale production, the volume was stepwise increased from 15 ml on day 1 to 220 ml on day 14, and a 16.8-fold expansion was achieved to 8.09 × 10^7^ total CAR-NK cells (Fig. 1d). Analysis of the CAR-NK cell product showed a NK cell purity of 99.59% in the Prodigy and 97.39% in the small-scale production (Fig. 1e). To quantify the contaminating T and B cells in the final product, a rare cell analysis would be necessary, requiring the acquisition of at least a total of 50 000 events in flow cytometry. Due to the low number of CAR-NK cells available for sampling in the SS cultures, the frequencies were not determined. In the PROD-CAR-NK cell product, overall, low frequencies were detected for other leukocytes, including T cells (0.05%), B cells (0.04%) and monocytes (0.00%). FACS plots of the CD3^+^ T cell population in the PROD-CAR-NK product compared to SS-produced cells are shown in Fig. 1f. NK cells were harvested and transported for 3 h in formulation solution on cooling pads from Bergisch-Gladbach to Frankfurt am Main where they were injected into OCI-AML2-xenografted mice. The overall duration from NK cell harvest until injection into mice was approximately 6 h. Subsequent flow-cytometry analysis revealed comparable cell surface expression of CD57 (3.8–9.2%), CD69 (94.2–97.6%), NKG2A (86.9–97.8%) and NKG2C (16.1–17.7%) on SS-UTD-, SS-CAR-, PROD-UTD- and PROD-CAR-NK cells (Fig. 1g). Furthermore, SS-CAR-NK and PROD-CAR-NK cells showed an increased cytotoxicity against CD33^+^ OCI-AML2 cells in a four-hour co-culture assay compared to SS-UTD- and PROD-UTD-NK cells at an unfavorable E:T-ratio of 1:5 (Fig. 1h).

Subsequently, the anti-leukemic effect of a single dose of PROD-CAR-NK cells was analyzed in an OCI-AML2 xenograft NOD.Cg-*Prkdc*^*scid*^ *Il2rg*^*tm1Wjl*^ Tg(CMV-IL3,CSF2,KITLG)1Eav/MloySzJ (NSG-SGM3) mouse model. NSG-SGM3 mice constitutively produce IL-3, GM-CSF, and stem cell factor, which reflects physiological conditions of a human host and promotes the engraftment of AML cells [28, 29]. NSG-SGM3 mice (male, 5–15 weeks old) received 0.5 × 10^6^ OCI-AML2 cells (GFP^+^, Luciferase^+^) intravenously followed by a single administration of 1 × 10^7^ SS-UTD-, SS-CAR- or PROD-CAR-NK cells at day 3 post tumor cell injection or remained untreated (UT). Additionally, on day 3, subcutaneous application of 25 000UI IL-2/mouse daily was started for mice which received NK cells (Fig. 1i). Mice which received no treatment quickly developed a systemic leukemia while treatment with SS-UTD-NK cells only resulted in a slight deceleration of leukemic cell growth. Administration of SS-CAR-NK as well as PROD-CAR-NK cells resulted in a strongly decelerated leukemic growth and reduced leukemic burden in treated animals to a similar degree (UT: *n* = 6, SS-UTD: *n* = 7, SS-CAR and PROD-CAR: *n* = 9; Fig. 1j–h).

**Fig. 2:**
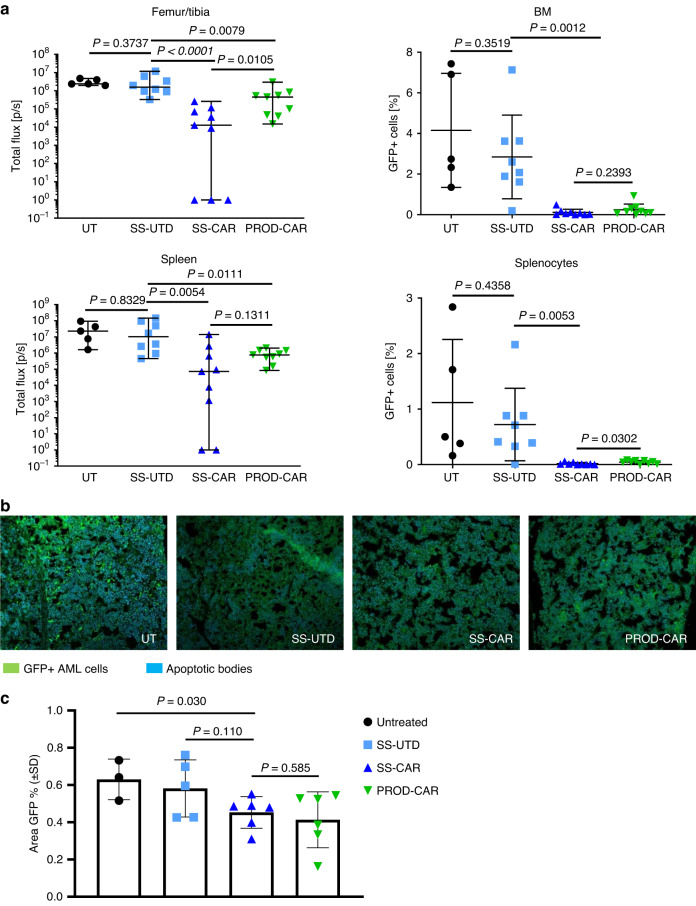
Small-scale- (SS-) produced and CliniMACS Prodigy® (PROD-) produced CD33-CAR-NK cells eradicate spleen- and BM-located AML cells in an OCI-AML2-xenograft NSG-SGM3 mouse model. **a** Total flux analysis of femurs/tibiae and spleens, as well as flow cytometry analysis of isolated cells from BMs or spleens at day 18 post AML-injection in untreated (UT), SS-untransduced (SS-UTD), or SS- and PROD-CAR-NK treated mice (*n* = 5–9 per group). Values of zero were set to 1 for total flux analysis. **b** Confocal microscopy images reveal infiltration of GFP^+^ OCI-AML2 cells (green) as well of apoptotic bodies (blue) in the BM of untreated animals. One representative image is shown for each group. **c** Quantification of the area of GFP^+^ cells in the confocal microscopy images. For total flux analysis median ± range is shown, and statistical analysis was performed by Mann–Whitney-test. For the rest mean ± SD is shown, and statistical analysis was performed by Student’s *t* test.

Bioluminescence imaging (BLI) analysis of femur, tibia, and spleen of mice on day 18 post OCI-AML2 cell administration revealed impeded AML cell engraftment in SS-CAR-NK and PROD-CAR-NK treated animals, which was confirmed by flow cytometry analysis of cells isolated from BM and spleen on day 19 (Fig. 2a). At that time SS- and PROD-CAR-NK treated animals showed full or near complete absence of GFP^+^ OCI-AML2 cells in these organs while human CD56^+^ NK cells were not detectable anymore (Supplementary Fig. 1a). Engraftment of GFP^+^ OCI-AML2 cells in the bone marrow as well as cell death and apoptotic bodies of BM stroma cells in untreated animals could be also observed in histological analyses via confocal microscopy. The presence of GFP^+^ as well as apoptotic cells was decreased to a similar degree following SS-CAR-NK and PROD-CAR-NK treatment (Fig. 2b, c). Overall, the application of NK and CAR-NK cells had no effect on the appearance, behavior, and weight of the animals. Analysis of peripheral blood at day one post (CAR-)NK cell application revealed no increased systemic levels of proinflammatory human cytokines secreted by adoptively applied NK cells (INF-γ, TNF-α, MIP-1α), while endogenous human GM-CSF production could be confirmed (Supplementary Fig. 1b).

## Discussion

NK cells possess a strong intrinsic killing capacity against malignant cells and can be applied in an allogeneic manner, which offers the potential for an off-the-shelf product and an alternative treatment option for CAR-T cell resistant malignancies. Hence, the development of novel processes for the generation of high amounts of CAR-NK cells under GMP conditions is crucial to facilitate clinical transition of CAR-NK cellular products. In this study, we were able to demonstrate that the automated production of CD33-targeting CAR-NK cells is possible and leads to a highly efficient functional cell product, which has the ability to eliminate AML cells in vivo. Automatically produced CD33-CAR-NK cells using a CliniMACS Prodigy^®^ device were similar to small-scale produced cells in terms of cell surface marker expression as well as in vitro and in vivo cytotoxicity against CD33^+^ OCI-AML2 cells. PROD-CAR-NK cells were not only able to strongly reduce leukemic burden in OCI-AML2-xenografted NSG-SGM3 mice but eradicated most of the BM- and spleen-located AML cells, while not persisting for more than two weeks in these organs. This might be crucial for the treatment of CD33^+^ AML without causing long-term myeloablation [30, 31]. Utilizing the CliniMACS Prodigy^®^, CAR-NK cells could be efficiently generated and cultivated for 14 days in an automated and closed system. During this process NK cells were stimulated with cytokines, which resulted in high abundances of CD69^+^ and NKG2A^+^ activated NK cells, and which was comparable to the small-scale process. After 14 days, a total number of 1.15 ×10^8^ CAR NK cells could be achieved in the Prodigy in comparison to 8.09 ×10^7^ CAR NK cells in small scale. Due to the automated large-scale procedure in the CliniMACS Prodigy^®^, high NK cell numbers could be initially isolated and cultivated, which lead to an overall higher NK cell yield in the final NK cell product. This is important, as fast generation of high numbers of CAR-NK cells in a standardized way under GMP conditions constitutes a critical step towards CAR-NK cell-product transition into clinics, and improvements of expansion efficiency in the CliniMACS Prodigy^®^ device could further accelerate the CAR-NK cell generation. Notably, CD33^+^ NK cells are reduced or eliminated in a CD33-CAR-NK cell culture which underlines the potential of this automated process which works even when fratricide-mediated elimination of CD33^+^ NK cells by CD33-targeting CAR-NK cells might pose a problem [18, 32]. Importantly, a three-hour transportation of cooled cells and subsequent three hours of preparation until injection into mice following harvest, did not abolish CAR-NK cells functionality. This shows that automated manufacturing of CAR-NK cells and subsequent transportation to a patient should work in principle if the CAR-NK cell production facility is not located at the clinic. Since the CliniMACS Prodigy^®^ device constitutes a closed and automated platform, it enables the production of GMP-compliant CAR-NK cells which could be ready to use in a clinical setting [33]. Overall, our data underline not only the feasibility of automated production, but also of transportation and application of a CD33-CAR-NK cell product in a preclinical in vivo setting, thereby demonstrating an impressive AML-targeting efficacy. In the future the automated CAR-NK cell production using the CliniMACS Prodigy^®^ device could be further scaled-up by the development of larger incubation chambers or by preparation of a master cell bank with Prodigy-generated CAR-NK cells as starting material for larger external bioreactors. In conclusion, this protocol has the potential to facilitate clinical production of primary CAR-NK cells for the treatment of leukemias as well as solid malignancies in the near future.

### Supplementary information

Supplementary-Figure 1

Supplementary-Figure Legend

## Data Availability

The raw data supporting the conclusion of this article will be made available by the authors, without undue reservation.
