# Supplementary figures and images for: Manufacturing of primary CAR-NK cells in an automated system for the treatment of acute myeloid leukemia
Source: Bone Marrow Transplant. 2024 Jan 22;59(4):489–95. doi: 10.1038/s41409-023-02180-4 (PMC10994833; doi:10.1038/s41409-023-02180-4)

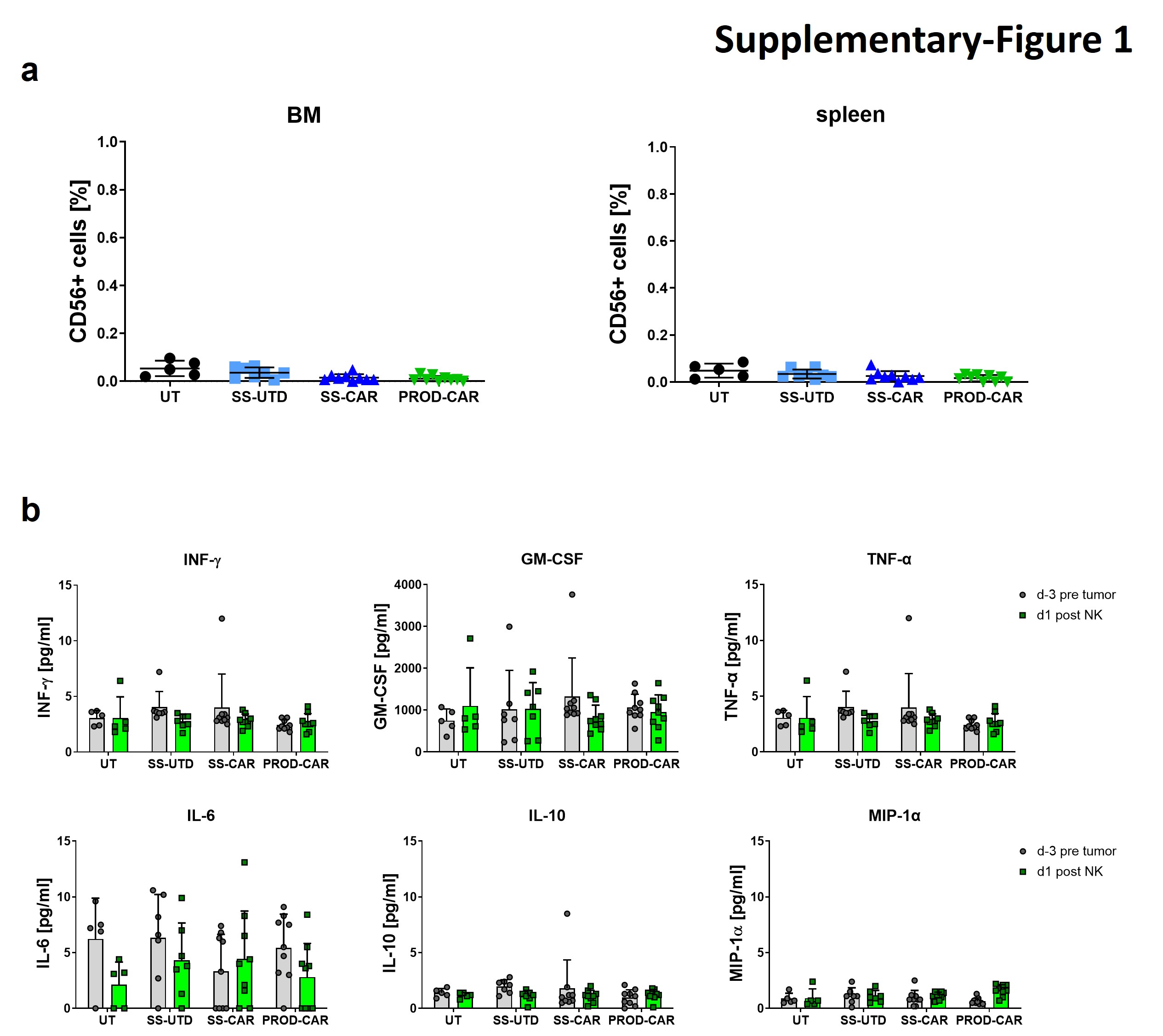

Supplement: Supplementary file 1 — Supplementary-Figure 1 [file 41409_2023_2180_MOESM1_ESM.jpg]
