# Supplementary material for: Manufacturing of primary CAR-NK cells in an automated system for the treatment of acute myeloid leukemia
Source: Bone Marrow Transplant. 2024 Jan 22;59(4):489–95. doi: 10.1038/s41409-023-02180-4 (PMC10994833; doi:10.1038/s41409-023-02180-4)
Supplement: Supplementary file 2 — Supplementary-Figure Legend [file 41409_2023_2180_MOESM2_ESM.docx]

Albinger et al.: **Manufacturing of primary CAR-NK cells in an automated system for the treatment of acute myeloid leukemia**

**Legends to supplementary figures**

**Supplementary-Figure 1**:

**a**: Flow cytometry analysis of CD56^+^ NK cells in isolated cells from BMs or spleens at day 18 post AML-injection in NSG-SGM3 mice (*n* = 5–9 per group). Mean ± SD. **b**: Serum analysis of pro-inflammatory human cytokines in blood isolated day 3 before AML injection and day 1 post NK cell application in NSG-SGM3 mice (*n* = 5–9 per group). Mean ± SD.
